# Reduced lung function and health-related quality of life after treatment for pulmonary tuberculosis in Gambian children: a cross-sectional comparative study

**DOI:** 10.1136/thorax-2022-219085

**Published:** 2022-09-15

**Authors:** Esin Nkereuwem, Schadrac Agbla, Azeezat Sallahdeen, Olumuyiwa Owolabi, Abdou K Sillah, Monica Genekah, Abdoulie Tunkara, Sheriff Kandeh, Maryama Jawara, Lamin Saidy, Andrew Bush, Toyin Togun, Beate Kampmann

**Affiliations:** 1 Vaccines and Immunity Theme, Medical Research Council Unit The Gambia at the London School of Hygiene & Tropical Medicine, Fajara, Gambia; 2 Faculty of Infectious and Tropical Diseases, London School of Hygiene & Tropical Medicine, London, UK; 3 Department of Health Data Science, University of Liverpool, Liverpool, UK; 4 Department of Infectious Diseases Epidemiology, London School of Hygiene & Tropical Medicine, London, UK; 5 Medical Research Council Unit The Gambia at the London School of Hygiene & Tropical Medicine, Fajara, Gambia; 6 Department of Paediatric Respiratory Medicine, National Heart & Lung Institute, Imperial College London - Royal Brompton Campus, London, UK; 7 Centre for Paediatrics and Child Health, Imperial College London, London, UK; 8 TB Centre, London School of Hygiene & Tropical Medicine, London, UK; 9 Vaccine Centre, London School of Hygiene & Tropical Medicine, London, UK

**Keywords:** Paediatric Lung Disaese, Respiratory Infection, Tuberculosis, Clinical Epidemiology

## Abstract

**Background:**

Post-tuberculosis (post-TB) lung disease is an under-recognised consequence of pulmonary tuberculosis (pTB). We aimed to estimate the prevalence of residual lung function impairment and reduced health-related quality of life (HRQoL) in children after pTB treatment completion.

**Methods:**

We conducted a cross-sectional comparative study of children aged less than 15 years at TB diagnosis who had completed treatment for pTB at least 6 months previously with a comparator group of age-matched children without a history of pTB. Symptoms, spirometry and HRQoL measured with PedsQL scale were collected. Variables associated with lung function impairment were identified through logistic regression models.

**Results:**

We enrolled 68 post-TB cases (median age 8.9 (IQR 7.2–11.2) years) and 91 children in the comparison group (11.5 (8.0–13.7) years). Spirometry from 52 (76.5%) post-TB cases and 89 (94.5%) of the comparison group met the quality criteria for acceptability and repeatability. Lung function impairment was present in 20/52 (38.5%) post-TB cases and 15/86 (17.4%) in the comparison group, p=0.009. Previous pTB and a history of chronic cough were significantly associated with the presence of lung function impairment (p=0.047 and 0.006 respectively). Forced expiratory volume in 1 s (FEV_1_), forced vital capacity (FVC) and FEV_1_/FVC *z*-scores were significantly lower in the post-TB cases compared with the comparison group (p= <0.001, 0.014 and <0.001, respectively). The distribution of the self-reported physical health score, and parent-reported physical, emotional, psychological, social and total HRQoL scores were significantly lower in the post-TB cases compared with the comparison group.

**Conclusions:**

Previous TB in children is associated with significantly impaired lung function and HRQoL.

**WHAT IS ALREADY KNOWN ON THIS TOPIC**

- Pulmonary tuberculosis (pTB) is associated with lung function impairment in adult TB survivors.

**WHAT THIS STUDY ADDS**

- Childhood pTB is associated with significantly increased odds of having impaired lung function, and significantly lower health-related quality of life scores, beyond 6 months after treatment completion.

**HOW THIS STUDY MIGHT AFFECT RESEARCH, PRACTICE OR POLICY**

- This suggests that a more holistic approach, which takes into account the physical and psychosocial effects of the disease, is needed to better define the outcomes of pTB treatment in children.

## Introduction

In 2020, an estimated 1.1 million children below 15 years developed tuberculosis (TB) worldwide, with paediatric disease accounting for approximately 11% of the 9.9 million new cases.^1^ The global annual numbers of TB cases in children have been on the rise, increasing from about 1 million in 2017 to 1.2 million in 2019, with the vast majority of these cases affecting the lungs.^1^

Traditionally, successful treatment for TB has been classified as either *cured* if there is no longer bacteriological evidence of TB in the last month of treatment, or *treatment completed* if the patient has clinically improved in the absence of bacteriological confirmation of cure at the end of treatment.^2^ Up to 85% of persons who are treated for the first TB episode will achieve treatment success.^3 4^ However, while emerging data derived almost exclusively from adult patients with TB suggest a high burden of residual morbidity after TB treatment, there is a huge knowledge gap regarding the post-TB outcomes in children.^5^

Many adult survivors who had pulmonary TB (pTB) develop chronic physical and psychosocial consequences such as persistently abnormal spirometry and reduced health-related quality of life (HRQoL).^5 6^ Adult pTB survivors have been shown to have twofold-to-fourfold higher odds of persistently abnormal spirometry compared with those without previous TB disease.^7–9^ Similarly, studies have documented persistence of respiratory symptoms, and reduced HRQoL despite successful completion of treatment for TB in adults.^10 11^ However, although a growing literature describes multiple post-TB morbidities in adults, the actual burden of each of these illnesses in children remains poorly described. More so, even though pTB accounts for at least 80% of all estimated TB cases that occur in children, post-TB lung disease (PTLD) remains an under-recognised health challenge in children, especially in regions of the world with high TB burden.^5 7^

Lung development begins in utero and continues into early adulthood before declining from about 20–25 years of age.^12 13^ Consequently, early insults to the lungs have been shown to affect lung growth and development adversely.^14^ This has the potential to accelerate decline in lung function, and increase risk of chronic respiratory illnesses in later life, with consequent reduction in the HRQoL.^12 15^ Several longitudinal studies have shown that lower respiratory tract infections (LRTI) during infancy are associated with reduced lung function in later childhood and adulthood.^16–19^ Furthermore, a longitudinal study in HIV-infected adolescents in South Africa suggested a correlation between prior history of TB or severe LRTI and decline in lung function.^20^

Based on these findings, we hypothesised that previously treated TB may be associated with persistently impaired lung function in children, and that children might experience similar or more severe adverse outcomes after completion of TB treatment. In this cross-sectional comparative study in The Gambia, we investigated the prevalence of residual lung function impairment in children after pTB treatment completion and compared their lung function and HRQoL to a comparison group of age-matched children without previous TB disease, who grew up in the same environment.

## Methods

### Study design

We conducted a cross-sectional comparative study at the childhood TB research clinic of the Medical Research Council Unit The Gambia at the London School of Hygiene & Tropical Medicine (MRCG at LSHTM), The Gambia. The MRCG at LSHTM has a long-standing collaboration with the Gambia National Leprosy and Tuberculosis Control Programme, and routinely contributes to the childhood TB diagnosis and case notification.^21–23^

### Post-TB cases

Using our childhood TB registry, we identified children who were diagnosed with confirmed or unconfirmed pTB at MRCG at LSHTM between January 2014 and December 2019 in the Greater Banjul Area (GBA).^23 24^ The GBA in The Gambia has a successful contact tracing and prophylaxis programme with a high isoniazid preventive treatment uptake of greater than 78%.^21^ The contact tracing programme provided the epidemiological framework for the recruitment of the post-TB cases and household comparison group in this study. These were children who had presented with symptoms suggestive of pTB following community-based contact tracing of sputum smear adults or following referral from peripheral health facilities. Unconfirmed TB was defined as presence of at least two of the following: symptoms and signs suggestive of TB, chest radiograph consistent with TB, close TB exposure and positive response to TB treatment; confirmed TB was defined by bacteriological confirmation of *Mycobacterium tuberculosis* (culture, Xpert MTB/RIF assay or both) from at least one respiratory specimen.^25^ At the time of TB diagnosis, from this cohort, we included children who were 15 years old or younger at the time of TB diagnosis and had successfully completed their anti-tuberculous therapy with a documented standard treatment outcome of *cured* or *treatment completed* not less than 6 months before the date of enrolment.^2^ In this paper, we have referred to these children as *post-TB cases*. Children were excluded if they were younger than 5 years old at enrolment (as they are often unable to perform spirometry reliably), unwilling to participate or if they had relocated from the study area. We also excluded children who were currently receiving treatment for recurrent pTB.

### Comparison group

We enrolled a comparison group which comprised children who were age-matched as closely as possible to the cases, and who lived in the same household as the cases but had never previously been diagnosed with pTB. We aimed to enrol at least two children into the comparison arm for each post-TB case enrolled from the household. In the context of this study, a household was defined as a group of individuals eating from the same pot and living in the same building.^26^ There were no children with signs and symptoms of active TB in the comparison group at the time of the study visit. We excluded all children with known chronic lung disease. All children who fulfilled the eligibility criteria for the comparison group were included in the study.

### Procedures

During a screening telephone call with the family, we invited all children who were eligible for enrolment as post-TB cases for a study visit. We also invited all other children in the same household, who met our enrolment criteria for the comparison group, to attend with the post-TB case. During the study visit, we obtained demographic and clinical information for each child. Clinical and laboratory data relating to previous TB disease were collected from the participants’ medical records at the MRCG at LSHTM childhood TB research clinic. We calculated anthropometric measurements, including height-for-age and body-mass-index-for-age *z*-scores using the WHO 2007 reference standards.^27^ Stunting was defined as a height-for-age *z*-score less than −2 SD for age and sex.

### Lung function measures

Lung function testing was performed on all study participants by a trained technician using the Easy on-PC portable spirometer (ndd Medical Technologies, Zurich, Switzerland). Spirometry was performed according to the American Thoracic Society (ATS)/European Respiratory Society guidelines.^28^ Before daily data collection, ambient temperature, humidity and altitude were recorded. Afterwards, the spirometer was calibrated using a 3 L syringe to ensure measured volumes within 3% of syringe volume. Briefly, up to eight forced exhalations were performed while sitting. The procedure was repeated for all participants after receiving a bronchodilator (BD), salbutamol via a spacer device. We included only spirometry traces that met the ATS quality criteria in the analysis. Each flow volume was reviewed for acceptability and repeatability by two independent reviewers who were unaware of the results from each other. In cases of discordance, the two clinicians agreed on a consensus result. The *z*-scores for the highest forced vital capacity (FVC) and forced expiratory volume in 1 s (FEV_1_) were recorded and used for analysis. Data were standardised using the Global Lung Initiative 2012 (GLI-2012) African American reference ranges.^29^ The GLI-2012 African American reference ranges have been previously validated among African children.^30^ Lung function impairment was defined as the presence of abnormal spirometry measurement classified as either obstructive, possible restriction or mixed obstruction–restriction pattern.^31^ Obstructive pattern was defined by a post-BD FEV_1_/FVC ratio below the lower limit of normal (LLN) for height, age and sex. Possible restriction was defined by post-BD FVC below the LLN for the height, age and sex, with a normal FEV_1_/FVC ratio.

### HRQoL

We measured the HRQoL using the generic PedsQL V.4.0 instrument.^32^ The PedsQL 4.0 has age-appropriate versions for children aged 2–18 years. It contains 23 items in four scales: physical health (8 items), emotional functioning (5 items), social functioning (5 items) and school functioning (5 items). A psychosocial health score (combined score of the emotional, social and school functioning subscales) and a total scale score can be computed. Items are scored on a 5-point Likert scale from 1 ‘Never a problem’ to 5 ‘Almost always a problem’, with a 1-week recall period. The responses are collected from the child and the parent independently, and answers are transformed into a 0–100 scale, with a higher score representing a better HRQoL. The PedsQL has been shown to have a high reliability and validity among children with chronic health conditions.^33 34^

The PedsQL instrument was in English Language. It was administered by staff who had been trained in its use and are proficient in English but also speak the local languages. Interviewers administered the instrument in any of the local languages that the subject felt most comfortable with. The children and their parents were interviewed separately.

### Statistical analysis

We used a convenience sample of all reachable and eligible post-TB cases from our childhood TB registry as described above, as well as all eligible children who were willing to be enrolled into the comparison group. Means (SD), medians (IQR) and proportions were used as appropriate to describe the burden of respiratory pathology using clinical and respiratory parameters, by prior TB status of the children. We assessed the association between prior TB status and those clinical and respiratory parameters using the mixed effects logistic and the Somers’ D statistic as appropriate to account for clustering between post-TB case and apparently healthy children within the same households. The Somers’ D statistic is a rank-based measure of association, that can accommodate clustered data.^35^ We investigated the association between prior TB status and abnormal lung function with a priori defined clinical data using univariable mixed effects logistic regressions. We then fitted a multivariable logistic regression model which incorporated variables with p values<0.20 in the univariable model. We also compared the distributions of the HRQoL scores between the post-TB cases and comparison group. Among the post-TB cases, we investigated the association between time since TB treatment completion, or the type of TB diagnosis, and abnormal lung function using the χ^2^ test for trend and Fisher’s exact test, respectively. ORs with 95% CI were reported. We investigated possible interaction between post-TB case and each of the covariates in the multivariable logistic regression, but no evidence of interaction was found. Data were analysed using Stata/SE V.17.0 (StataCorp, College Station, Texas, USA).

### Role of funding source

The funder of the study had no role in study design, data collection, data analysis, data interpretation or writing of the report. The corresponding author had full access to all study data and had final responsibility for the decision to submit for publication.

## Results

### Participant characteristics

**Figure 1:**
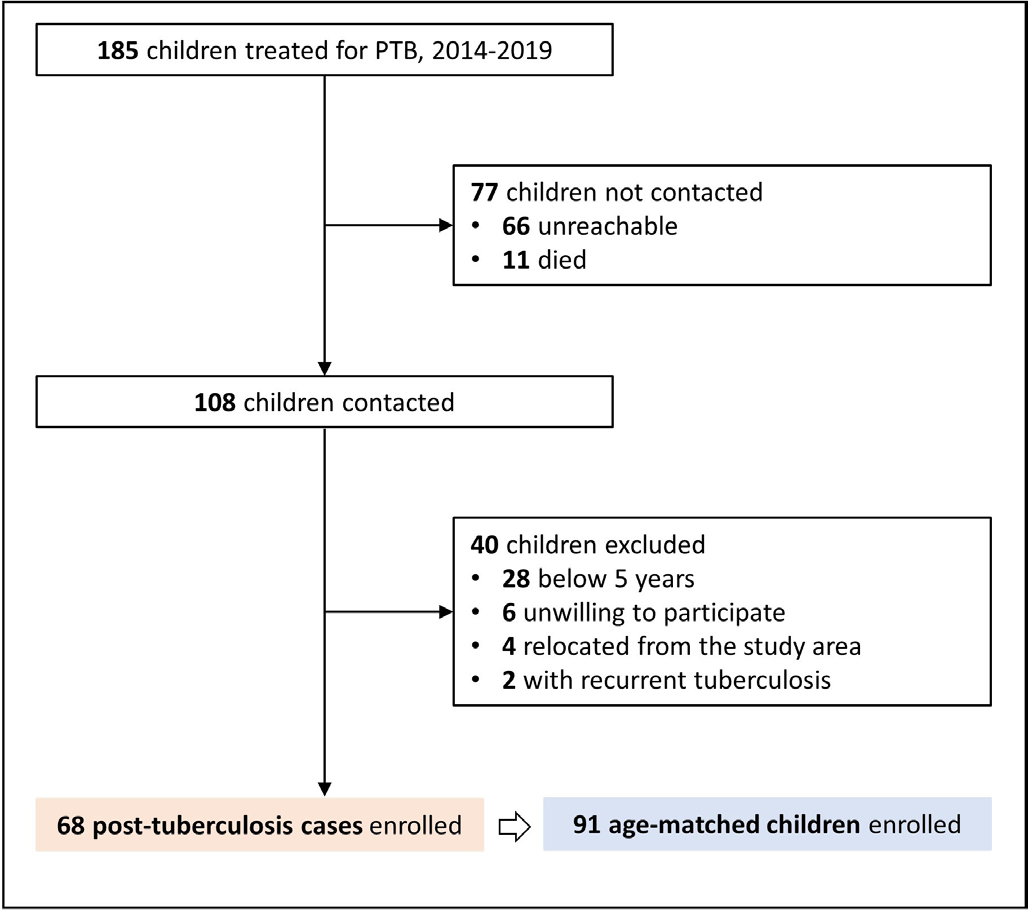
Study flowchart for selection of study participants. PTB, pulmonary TB.

A total of 185 children were identified from the childhood TB registry to have been diagnosed and treated for pTB from 2014 to 2019. Of these, families of 108 children were contacted, of whom 40 children were ineligible for enrolment because they were below 5 years of age (n=28), unwilling to participate (n=6), had relocated from the study area (n=4) or were ill with a recurrent pTB (n=2). Overall, 68 post-TB cases and 91 children in the comparison group were enrolled between 13 January 2020 and 3 March 2021 (figure 1). The number of children enrolled as post-TB cases and comparison group from each household are shown in online supplemental material table 1.

10.1136/thorax-2022-219085.supp1 Supplementary data

The demographic characteristics of the study participants are detailed in table 1. The post-TB cases had a median age of 8.9 years (IQR 7.2–11.2) compared with 11.5 years (IQR 8.0–13.7) in the comparison group, which was statistically significant but thought not to be clinically important. The median age at TB diagnosis was 6.5 years (IQR 3.7–9.3), and the median duration since completion of TB treatment was 19.2 months (IQR 10.2–44.4). A higher proportion of the post-TB cases were stunted compared with the comparison group (13/68, 19.1% in post-TB cases vs 6/91, 6.6% in comparison group, p=0.032). A history of non-TB LRTI in the preceding 12 months was more commonly reported in the post-TB cases than in the comparison group (6/68, 8.8% in the post-TB cases vs 1/91, 1.1% in the comparison group, p=0.036). The prevalence of HIV infection among the post-TB cases was 13.2%. Three (4.4%) post-TB cases had been previously diagnosed with asthma. There were no children living with HIV or asthma in the comparison group. We found no significant differences in the distribution of exposure to environment tobacco smoke or household biomass smoke exposure between post-TB cases and the comparison group (table 1).

**Table 1:** Participant characteristics, stratified by prior tuberculosis status

	Post-TB cases (n=68)	Comparison group (n=91)	P value
Age, years, median (IQR)	8.9 (7.2–11.2)	11.5 (8.0–13.7)	0.001*
Age at TB diagnosis, years, median (IQR)	6.5 (3.7–9.3)	–	–
Time since TB treatment completion, months, median (IQR)	19.2 (10.2–44.4)	–	–
Sex			
Female	32 (47.1)	34 (37.4)	0.259†
Male	36 (52.9)	57 (62.6)	
BCG scar present	60 (90.9)	82 (92.1)	0.789†
Stunted	13 (19.1)	6 (6.6)	0.032†
Underweight	17 (25.0)	19 (20.9)	0.521†
Comorbidities			
HIV infection	9 (13.2)	0	0.002†
Asthma	3 (4.4)	0	0.081†
Non-TB LRTI in preceding 12 months	6 (8.8)	1 (1.1)	0.036†
Allergies	6 (8.8)	4 (4.4)	0.196†
Exposure to environmental tobacco smoke	25 (36.8)	30 (33.0)	0.563†
Household biomass smoke exposure	66 (97.1)	89 (97.8)	0.333†
Type of TB diagnosis			
Confirmed	24 (35.3)	–	–
Unconfirmed	44 (64.7)	–	–

Data are presented as n (%) unless otherwise indicated.

*P value based on the non-parametric Somers’ D measure, accounting for clustering.

†P values obtained from fitting a univariable mixed effect logistic regression where prior TB status is the dependent variable.

LRTI, lower respiratory tract infection; TB, tuberculosis.

### Chronic respiratory symptoms and HRQoL

More than half (35/68, 51.5%) of the post-TB cases reported one or more recurrent respiratory symptom(s) in the preceding 6 months compared with about one-third (34/91, 37.4%) of the comparison group. The most common reported symptom in both groups was cough (21/68, 30.9% in the post-TB cases and 20/91, 22.0% in the comparison group). Furthermore, the post-TB cases were more likely to report failure to gain weight compared with the comparison group (19/68, 27.9% in the post-TB cases vs 13/91, 14.3% in the comparison group, p=0.026, table 2).

**Table 2:** Clinical and respiratory parameters, stratified by prior tuberculosis status

	Post-tuberculosis cases	Comparison group	P value
Self-reported clinical and respiratory symptoms*	n=68	n=91	
Cough, n (%)	21 (30.9)	20 (22.0)	0.176†
Sputum, n (%)	8 (11.8)	7 (7.7)	0.397†
Wheeze, n (%)	6 (8.8)	9 (9.9)	0.819†
Easy fatigability, n (%)	15 (22.1)	10 (11.0)	0.095†
Chest pain, n (%)	17 (25.0)	15 (16.5)	0.105†
Failure to gain weight, n (%)	19 (27.9)	13 (14.3)	0.026†
Any respiratory symptom, n (%)	35 (51.5)	34 (37.4)	0.068†
Child self-report quality of life	n=63	n=89	
Physical health, median % (IQR)	68.8 (56.3–93.8)	81.3 (62.5–100)	0.016‡
Emotional functioning, median % (IQR)	80.0 (60.0–90.0)	80.0 (60.0–80.0)	0.538‡
Social functioning, median % (IQR)	90.0 (80.0–100)	90.0 (80.0–100)	0.333‡
School functioning, median % (IQR)	70.0 (60.0–90.0)	80.0 (60.0–90.0)	0.221‡
Psychosocial health, median % (IQR)	80.0 (70.0–86.7)	80.0 (70.0–90.0)	0.676‡
Total score, median % (IQR)	73.9 (65.2–89.1)	78.3 (67.4–89.1)	0.215‡
Parent-report quality of life	n=63	n=82	
Physical health, median % (IQR)	87.5 (68.8–93.8)	100 (87.5–100)	<0.001‡
Emotional functioning, median % (IQR)	80.0 (70.0–100)	90.0 (80.0–100)	0.001‡
Social functioning, median % (IQR)	100 (90.0–100)	100 (100–100)	0.019‡
School functioning, median % (IQR)	80.0 (60.0–100)	80.0 (60.0–100)	0.347‡
Psychosocial health, median % (IQR)	73.3 (80.0–93.3)	90.0 (80.0–96.7)	0.004‡
Total score, median % (IQR)	82.6 (71.7–93.5)	91.3 (82.6–97.8)	<0.001‡
Spirometry	n=52	n=86	
FEV_1_ *z*-score, mean (SD)	−1.52 (−0.99)	−0.83 (−0.84)	<0.001‡
FVC *z*-score, mean (SD)	−1.32 (1.02)	−0.87 (0.89)	0.014‡
FEV_1_/FVC ratio *z*-score, mean (SD)	−0.54 (0.91)	−0.03 (0.81)	0.001‡
Abnormal spirometry, n (%)	20 (38.5)	15 (17.4)	0.009†
Pattern of spirometry			
Normal, n (%)	32 (61.5)	71 (82.6)	0.029§
Obstructive, n (%)	1 (1.9)	2 (2.3)	
Restrictive, n (%)	19 (36.4)	13 (15.1)	

*Question read as follows: ‘Have you had any of the following occurring often or repeatedly in the last 6 months?’.

†P values obtained from fitting a univariable mixed effect logistic regression where prior TB status is the independent variable.

‡P value based on the non-parametric Somers’ D measure, accounting for clustering.

§P values obtained from fitting a univariable mixed effect logistic regression where prior TB status is the dependent variable.

FEV1, forced expiratory volume in 1 s; FVC, forced vital capacity; TB, tuberculosis.

Post-TB cases had lower median percentage scores on the self-reported physical functioning scale of the PedsQL (68·8%) compared with the comparison group (81.3%), p=0.016. More so, the post-TB cases had lower median percentage scores compared with the comparison group in five out of six of the parent-reported PedsQL scales (table 2).

### Lung function impairment

The spirometry data from 52 (76.5%) post-TB cases and 86 (94.5%) of the comparison group met the quality criteria for acceptability and repeatability. The mean *z*-scores for the FEV_1_, FVC and FEV_1_/FVC ratio were significantly lower in the post-TB cases compared with the comparison group (p<0.001, p=0.014 and p=0.001, respectively). The proportion of children with impaired lung function was significantly higher in the post-TB cases (20/52, 38.5%) compared with the comparison group (15/86, 17.4%), p=0·009. Restrictive pattern abnormalities were the most common in both post-TB cases (19/52, 36.4%) and comparison group (13/15, 15.1%). There were no study participants with mixed obstructive–restrictive pattern (table 2).

### Factors associated with lung function impairment

Compared with the comparison group, there was strong evidence for abnormal lung function in post-TB cases, after controlling for prespecified sociodemographic and clinical covariates (adjusted OR (aOR) 3.9, 95% CI 1.1 to 15.1, p=0.047, table 3). A self-reported history of frequent or repeated cough in the preceding 6 months was found to be associated with abnormal lung function among the children in this study (aOR 19.0, 95% CI 1.6 to 226.0, p=0·006).

**Table 3:** Factors associated with lung function impairment in all children

	Univariable analysis		Multivariable analysis	
	OR (95% CI)	P value	aOR (95% CI)	P value
Post-TB case	4.2 (1.5 to 12.0)	0.009	3.9 (1.1 to 15.1)	0.047
Age ≥10 years	2.4 (0.8 to 7.9)	0.516	3.0 (0.7 to 12.1)	0.121
Male	1.0 (0.3 to 3.5)	0.915	0.7 (0.2 to 2.2)	0.504
BCG scar present	3.1 (0.4 to 25.7)	0.291	–	–
Stunted	11.0 (1.3 to 92.0)	0.001	4.3 (0.6 to 31.4)	0.147
Underweight	5.7 (1.2 to 27.1)	0.004	3.2 (0.9 to 12.2)	0.080
Comorbidities				
HIV infection	17.0 (1.9 to 151.2)	0.016	2.0 (0.1 to 49.6)	0.671
Asthma	1.5 (0.1 to 16.9)	0.739	–	–
Non-TB LRTI in preceding 12 months	1.5 (0.3 to 8.6)	0.634	–	–
Allergies	1.5 (0.4 to 6.4)	0.574	–	–
Respiratory symptoms*				
Cough	4.2 (1.1 to 15.8)	0.002	19.0 (1.6 to 226.0)	0.006
Sputum	3.2 (0.5 to 20.5)	0.195	0.1 (0.1 to 1.9)	0.137
Wheeze	1.1 (0.2 to 7.2)	0.902	–	–
Easy fatigability	2.6 (0.7 to 10.1)	0.207	–	–
Chest pain	1.9 (0.4 to 8.6)	0.009	4.7 (0.8 to 29.4)	0.096
Failure to gain weight	3.6 (1.5 to 9.0)	0.011	7.1 (0.8 to 66.9)	0.087
Any respiratory symptom	2.8 (0.7 to 11.6)	0.014	0.1 (0.01 to 1.3)	0.094
Exposure to environmental tobacco smoke	1.1 (0.1 to 13.3)	0.935	–	–

Data are presented as n (%) unless otherwise indicated.

*Question read as follows: ‘Have you had any of the following occurring often or repeatedly in the last 6 months?’.

aOR, adjusted OR; LRTI, lower respiratory tract infections; TB, tuberculosis.

Among the post-TB cases, there was no evidence of association between time since TB treatment completion, or the type of TB diagnosis, and presence of abnormal lung function (table 4).

**Table 4:** Time since tuberculosis treatment and type of tuberculosis diagnosis, stratified by spirometry outcome in post-tuberculosis cases

	Normal spirometry (n=32)	Abnormal spirometry (n=20)	P value
Time since TB treatment completion (months)			
<12 months	11 (34.4)	7 (35.5)	0.878*
12 to <24 months	9 (28.1)	4 (20.0)	
>24 months	12 (37.5)	9 (45.0)	
Type of TB diagnosis			
Confirmed	13 (40.6)	7 (35.0)	0.774†
Unconfirmed	19 (59.4)	13 (65.0)	

*P value obtained from Cochran-Armitage test for trend.

†P value obtained from Fisher’s exact test.

TB, tuberculosis.

## Discussion

We compared the lung function measurements and HRQoL between former childhood TB cases who were at least 6 months post-treatment completion and a comparison group of age-matched children who had never been previously diagnosed with pTB disease and lived in the same household. The post-TB cases had more than threefold increased odds of lung function impairment, predominantly of the restrictive type, compared with the comparison group. Similarly, post-TB cases had significantly lower self-reported physical HRQoL scores when compared with the comparison group.

To our knowledge, this is the first study describing the prevalence and pattern of lung function impairment associated with pTB treatment completion in children. The finding of a high burden of post-TB lung function impairment is consistent with findings in adults from previous literature which suggest that pTB survivors had twofold-to-fourfold increased odds of abnormal spirometry compared with healthy controls.^7–9^ Our findings are similar to data in adolescents living with HIV which showed that prior pTB was associated with low FEV_1_ and FVC, suggesting a similar or possibly higher population burden of these post-TB pulmonary sequelae in children.^20^

Published evidence in adolescents has shown that the effect of HIV on lung function was reduced after adjusting for previous pTB infection.^20^ Similarly, adults living with HIV have been found to have no different or less extensive lung damage following TB compared with HIV-negative adults, even when they have had similar duration of TB.^10 36^ Likewise, in our study, HIV infection was not significantly associated with lung function impairment among all children. These findings suggest that pTB may be associated with reduced lung function, even in people living with HIV. However, we acknowledge that the small number of children living with HIV among the sample population (with none in the comparison group) suggest that these findings need to be interpreted with caution.

The substantial burden of chronic respiratory symptoms and abnormal lung function (prevalence 17.4%) among the apparently healthy comparison group in this study is alarming. This is possibly due to the potential effect of frequent respiratory infections, or exposure to environmental factors such as tobacco smoke and biomass smoke exposure on lung function in children. Evidence in the literature has documented abnormal spirometry in as high as 16.5% of children aged 6–8 years who were exposed to open fire cooking.^37^ Similarly, early-life LRTI has been shown to be associated with impaired lung function later in life.^19^ Our findings warrant further investigation to explore the prevalence and risk factors of impaired lung function in the apparently-healthy population, who nevertheless experiences frequent occurrences of early-life LRTI and a high level of exposure to biomass smoke in the household due to cooking on open fires.^38^

Our data show that pTB is associated with a reduction in all domains of the HRQoL in the longer term. In the present study, the post-TB cases self-reported primarily impaired physical functioning, while their parents reported an impairment across five out of six HRQoL domains. Adverse physical and psychosocial consequences are well recognised outcomes of pTB.^39^ While studies have shown that HRQoL improves during standard treatment for TB,^40–43^ our data suggest that many children may have reduced HRQoL long after treatment completion, although we only report a snapshot, given that we have no pre-TB measurements. Moreover, the discrepancy between the self-reported and parent-reported HRQoL scores are not unexpected. Parents and caregivers are known to perceive their child’s ailment as more problematic.^34^ Our data support the need to assess the HRQoL as an outcome measure after treatment completion and beyond, preferably using a more qualitative approach.^40^

The combination of spirometry and HRQoL measures as tools to assess health and well-being after TB treatment represents a strength of this study, as this further highlights the association between previous TB and long-term physical and psychosocial well-being.^44^ A limitation of our study is that spirometry alone cannot be relied on to make inference about the presence of restrictive lung function abnormalities. This often requires diagnostic confirmation by measuring the total lung capacity, which was not possible in our setting.^31^ Second, as the lung function was not assessed prior to TB disease, we cannot currently confirm a causal relationship between the disease and impaired lung function, although further prospective work is currently ongoing. Third, a single spirometry measurement may not fully reflect the evolution of the lung function, which has been shown to change over time after treatment completion for TB.^10^ This is especially so in children whose lungs are still undergoing developmental changes.^12 13^ Fourth, our participant recruitment required prior attendance of the MRCG at LSHTM childhood TB research clinic, with a potential for selection bias away from those who were diagnosed elsewhere.

In conclusion, previous pTB in children was associated with reduced spirometric *z*-scores, and HRQoL scores compared with age-matched peers without a history of TB. Post-TB cases had greater than threefold increased odds of having abnormal lung function compared with the comparison group. Longitudinal studies to further characterise the evolution of symptoms and lung volumes after TB treatment completion in children are needed to help define and further characterise PTLD in children. Finally, we recommend a more holistic approach to define TB treatment outcome which considers the evaluation and management of sequelae, especially in children, to improve health and well-being across the life course. Anti-tuberculous chemotherapy leads to a bacteriological cure, but is far from the complete answer to tuberculous disease in children.

## Data Availability

Data are available upon reasonable request.
