# Supplementary material for: Reduced lung function and health-related quality of life after treatment for pulmonary tuberculosis in Gambian children: a cross-sectional comparative study
Source: Thorax. 2022 Sep 15;78(3):281–7. doi: 10.1136/thorax-2022-219085 (PMC9985734; doi:10.1136/thorax-2022-219085)
Supplement: Supplementary data [file thorax-2022-219085supp001.pdf]

Supplementary material

Number of post-TB cases and comparison group children enrolled from each household

Supplementary Table 1 shows the number of post-tuberculosis (post-TB) enrolled along with the corresponding number of age-matched comparison group children from each household. Household 45 had two post-TB cases, and there were 13 households which did not have an eligible age-matched child for the comparison group.

S Table 1: Post-TB cases and age-matched comparison group children from each household

Household match ID	Post-TB cases (n=68)	Comparison group (n=91)	Total (n=159)
1	1	2	3
2	1	3	4
3	1	2	3
4	1	1	2
5	1	1	2
6	1	3	4
7	1	1	2
8	1	3	4
9	1	1	2
10	1	3	4
11	1	3	4
12	1	3	4
13	1	3	4
14	1	1	2
15	1	1	2
16	1	1	2
17	1	1	2
18	1	1	2
19	1	2	3
20	1	1	2
21	1	0	1
22	1	0	1
23	1	0	1
24	1	0	1
25	1	2	3
26	1	3	4
27	1	1	2
28	1	0	1
29	1	0	1
30	1	3	4
31	1	1	2
32	1	1	2
33	1	0	1
34	1	1	2

Nkereuwem E, *et al. Thorax* 2023; 78:281–287. doi: 10.1136/thorax-2022-219085

35	1	2	3
36	1	2	3
37	1	0	1
38	1	0	1
39	1	2	3
40	1	0	1
41	1	1	2
42	1	2	3
43	1	1	2
44	1	3	4
45	2	3	5
46	1	1	2
47	1	1	2
48	1	2	3
49	1	1	2
50	1	2	3
51	1	1	2
52	1	2	3
53	1	0	1
54	1	1	2
55	1	2	3
56	1	0	1
57	1	2	3
58	1	0	1
59	1	2	3
60	1	1	2
61	1	2	3
62	1	1	2
63	1	1	2
64	1	1	2
65	1	1	2
66	1	1	2
67	1	1	2

Nkereuwem E, *et al. Thorax* 2023; 78:281–287. doi: 10.1136/thorax-2022-219085

Distribution of spirometry z-scores across age

Supplementary Figures 1-3 show the scatterplots for spirometry (FVC, FEV_1, and FEV_1/FVC ratio) z-scores across age, by post-tuberculosis cases and comparison group. Scatterplots for spirometry z-scores did not show any linear trend. The spread of *z*-scores was less variable for the FEV1/FVC ratio compared to FVC and FEV_1 *z*-scores across age. A greater proportion of *z*-scores for the post-tuberculosis cases were distributed below the lower threshold value of –1.64, compared to the comparison group (Fig. 1-3).

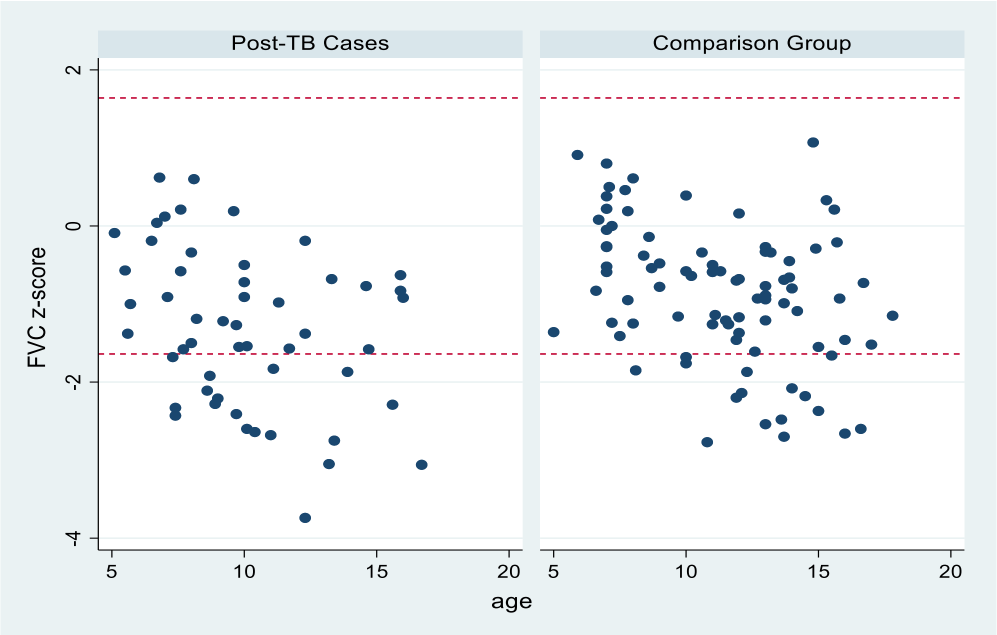

S Figure 1: Scatterplots for GLI_{2012} *z*-scores for FVC. Plots also demonstrate the distribution of the z-score values around 1·64 (Upper Limit of Normal) and −1·64 (Lower Limit of Normal)

Nkereuwem E, *et al. Thorax* 2023; 78:281–287. doi: 10.1136/thorax-2022-219085

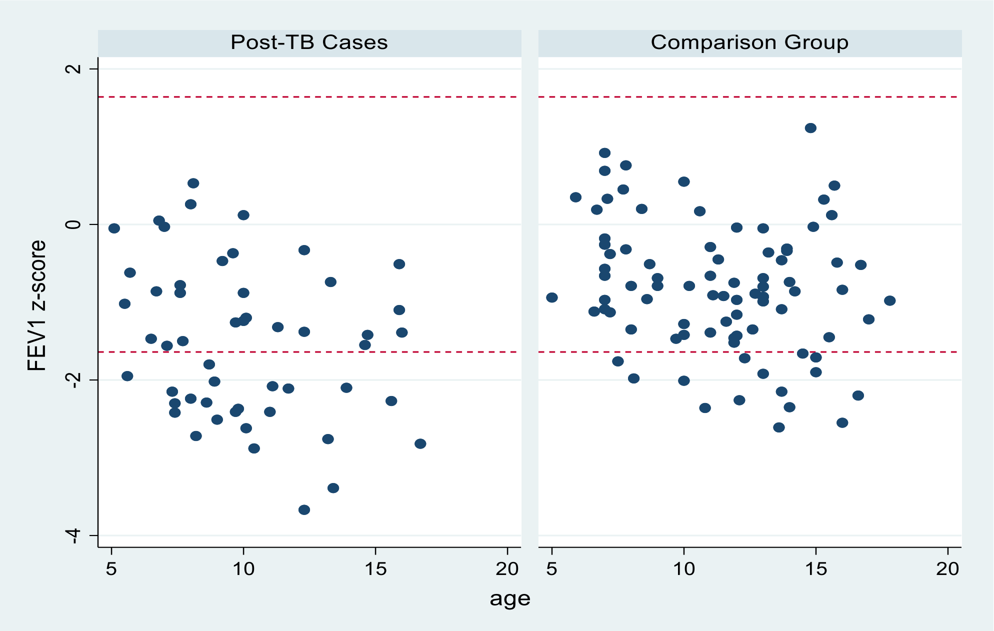

S Figure 2: Scatterplots for GLI_{2012} *z*-scores for FEV_1. Plots also demonstrate the distribution of the z-score values around 1·64 (Upper Limit of Normal) and −1·64 (Lower Limit of Normal)

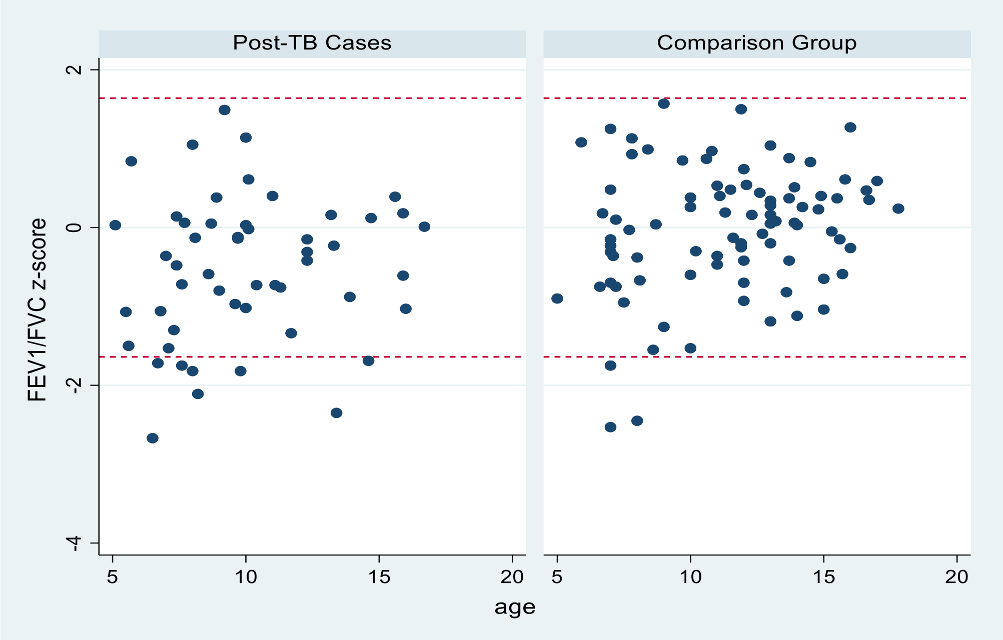

S Figure 3: Scatterplots for GLI_{2012} *z*-scores for FEV_1/FVC. Plots also demonstrate the distribution of the z-score values around 1·64 (Upper Limit of Normal) and −1·64 (Lower Limit of No

Nkereuwem E, *et al. Thorax* 2023; 78:281–287. doi: 10.1136/thorax-2022-219085
